# Supplementary figures and images for: Genome-Wide Identification of MYC Transcription Factors and Their Potential Functions in the Growth and Development Regulation of Tree Peony (Paeonia suffruticosa)
Source: Plants (Basel). 2024 Feb 2;13(3):437. doi: 10.3390/plants13030437 (PMC10857424; doi:10.3390/plants13030437)

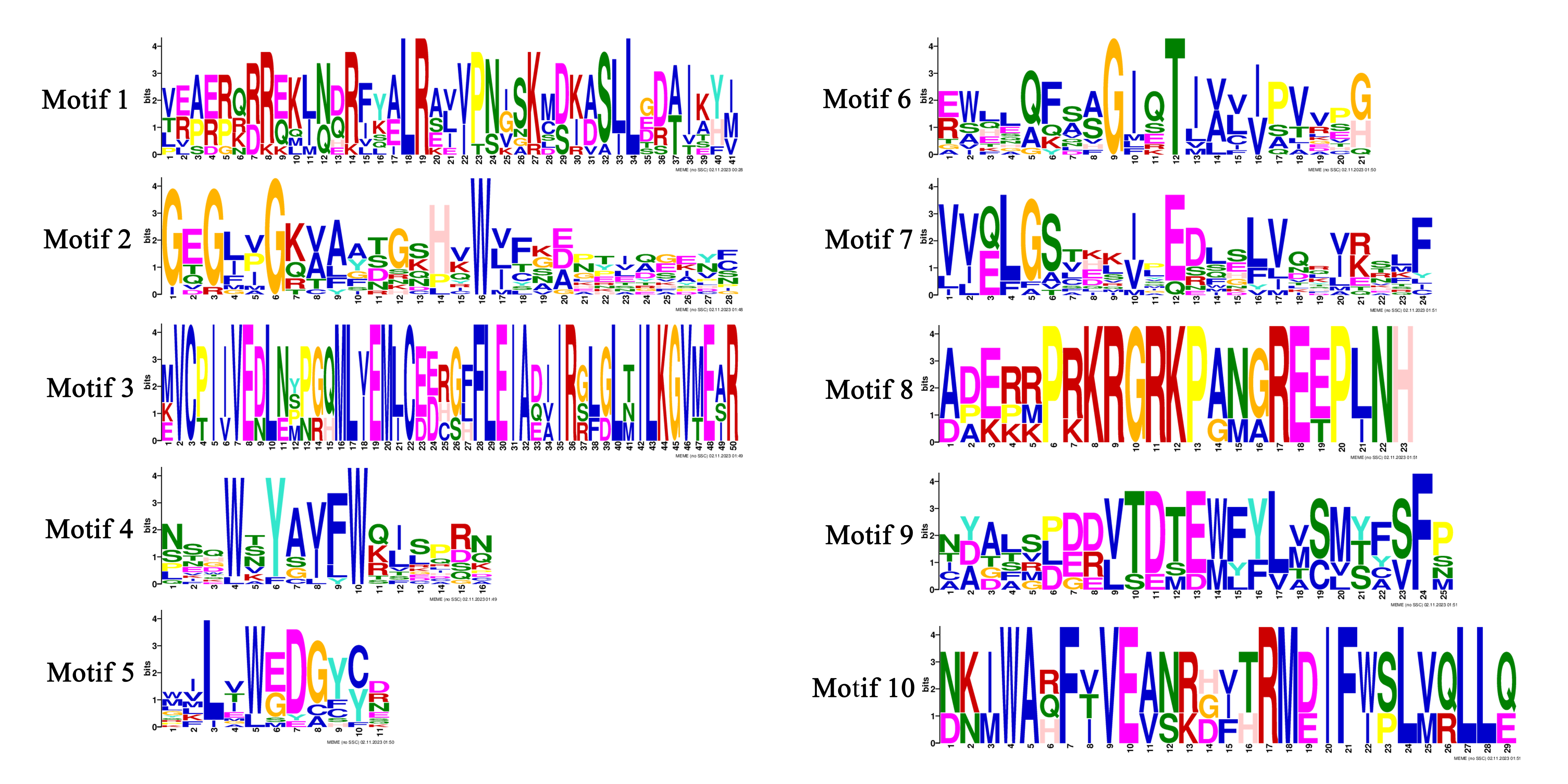

Supplement: Supplementary file 1 [file plants-13-00437-s001.zip › Supplementary Figure S1. The sequence of top ten conserved motifs in 15 PsMYCs.tif]

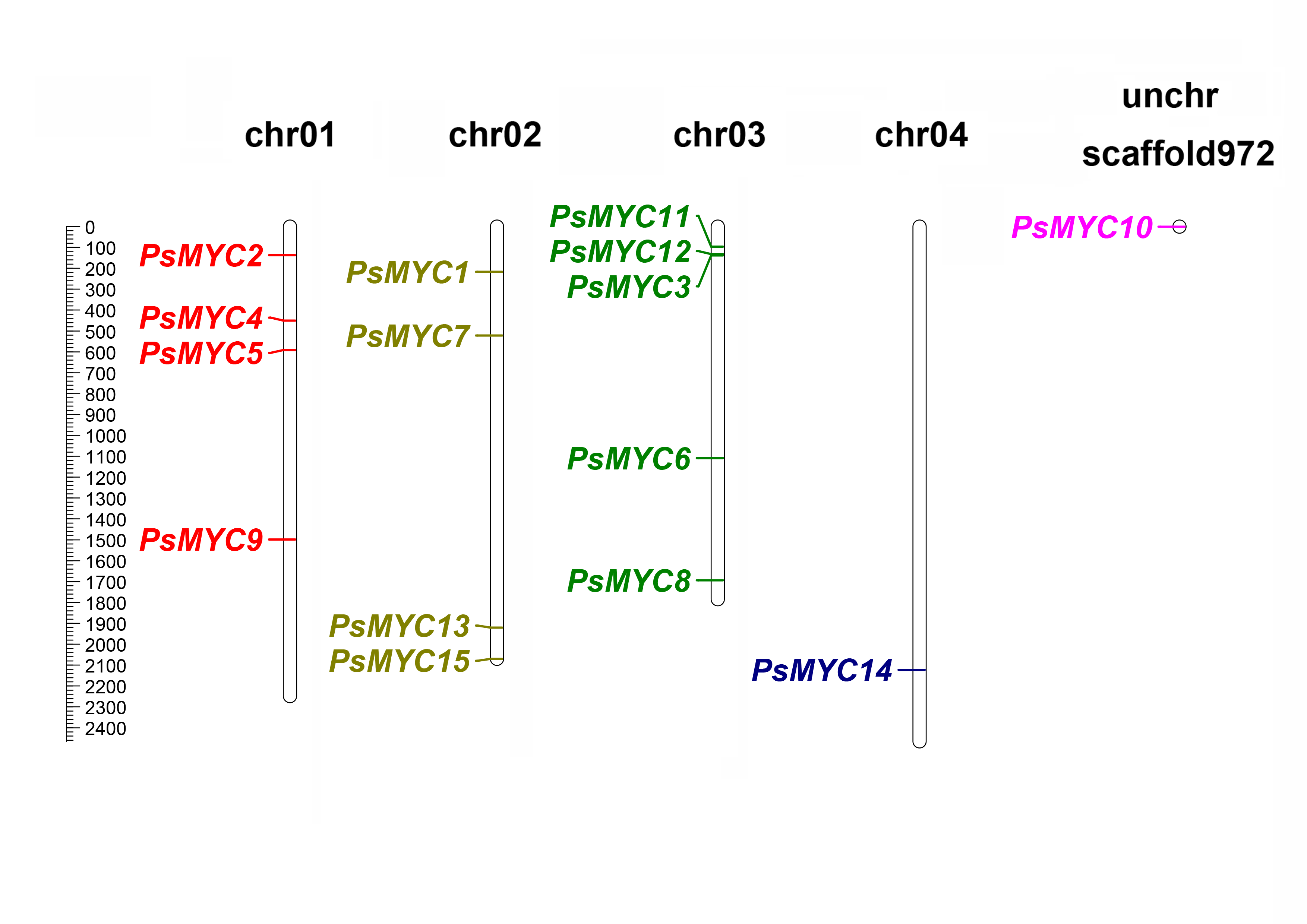

Supplement: Supplementary file 1 [file plants-13-00437-s001.zip › Supplementary Figure S2. The chromosomal location analysis of 15 PsMYCs.tif]
